# Policy Uncertainty, Official Social Capital, and the Effective Corporate Tax Rate—Evidence From Chinese Firms

**DOI:** 10.3389/fpsyg.2022.899021

**Published:** 2022-05-18

**Authors:** Long Wang, Dong Yang, Dongdong Luo

**Affiliations:** ^1^School of Economics, Peking University, Beijing, China; ^2^School of Statistics, Southwestern University of Finance and Economics, Chengdu, China

**Keywords:** policy uncertainty, official social capital, effective tax rate, official promotion, sustainable development

## Abstract

The political environment has a significant impact on the sustainable development of enterprises. This manuscript aims to investigate the effect of policy uncertainty and official social capital on enterprises’ effective tax rate (ETR) due to the change of officials. Based on the panel data from the Chinese Industrial Enterprise Database from 1998 to 2009, it is shown that the policy uncertainty caused by the change of local government officials significantly increases the ETR of enterprises. Meanwhile, municipal officials who have social ties with provincial officials in their province also tend to raise the ETR of industrial enterprises, and this tendency is more evident when the officials take office. Further research shows that the effects vary in many aspects for policy uncertainty and social capital on the ETR of enterprises. The findings of this manuscript provide support for a deeper understanding of the change in local government fiscal policies and give suggestions to strengthen political environmental governance for the sustainable development of enterprises.

## Introduction

New institutional economists, represented by Douglas North, have emphasized the influence of the institutional environment on the sustainable development of enterprises. One of them is the region’s political environment, especially the policy uncertainty due to the change of government officials. It has shown that when facing pressure for promotion, officials will stimulate macroeconomic growth in various possible ways to achieve their own promotion goals, and the policy uncertainty can have a significant impact on the sustainable development of enterprises (Jones and Olken, 2005; Li and Zhou, 2005; Kahn et al., 2015; Deng et al., 2019; Yu and Mai, 2020; Chen, 2021). In China, various policies such as administrative approval, land acquisition, loan guarantees, and tax exemptions are in the hands of local governments, and the continuity of local government economic and fiscal policies has an essential impact on the development of enterprises and the economy. The government-led economy has been an important feature of China’s economic development since the Reform and Opening in 1978. As the government’s legal representatives, local officials play a crucial role in the region’s economic growth. Local governments can promote their economic goals through various means, such as promoting infrastructure construction, increasing public services, and adopting specific fiscal measures to affect the sustainable development of enterprises (Earle and Gehlbach, 2015; Ma et al., 2018; He et al., 2019; Chen et al., 2021; Akhtari et al., 2022). Although this economic development mode dominated by government officials has created high economic growth, it has also brought many drawbacks. One of the main problems is policy uncertainty caused by changes of local officials. As the core government officials change, the former officials’ economic and regional development policies are at risk of changing during their tenure. The stable government-enterprise relationship and political-enterprise ecology are also likely to be affected by the new officials’ new “rules of the game” (Hardouvelis et al., 2018; Cao et al., 2019; Chen et al., 2020). At the same time, it is also common for some local government leaders in China to be transferred frequently, with their terms of office not expiring. Such frequent changes in officials exacerbate the discontinuity of local policies and lead to increased risks for enterprises (Pástor and Veronesi, 2013; Chen et al., 2018). Therefore, one of the hypotheses of this manuscript is that policy uncertainty arising from changes in officials may largely influence the fiscal policy choices of local governments, which in turn affects the sustainable development of enterprises.

Meanwhile, researchers have been increasingly interested in social capital theory. It has been treated as another type of capital besides physical capital and human capital, which plays a vital role in the economic behavior of actors (Putnam, 1993; Stone, 2001; Robison et al., 2002; Kawachi et al., 2008; Muringani et al., 2021). Social capital is rationally seen as a social resource and mobilized to serve its development. It manifests itself as a network of social relations, but unlike what is generally thought of as displaceable social relations, such as relations of production, administrative authorization, etc. It is a network of social connections, such as place of origin, first place of work, first school of education, etc. It has been widely known that China is a society that emphasizes interpersonal relationships and social networks (Kong, 2011; Wang and Kanungo, 2020). Thus, this social capital is presented in the form of social ties, such as kinship, friends, classmates, fellow villagers, neighbors, membership in a particular clique, or party (Borgatti et al., 1998; Neumeyer et al., 2019; Gannon and Roberts, 2020). In China, during the transition period, the government further influences enterprise behavior through economic policies such as corporate taxation to achieve the government’s goals, which is also proved by a growing number of studies (Pan et al., 2015; Yang et al., 2015; Cull et al., 2017; Guo and Shi, 2018; Chen et al., 2020). Meanwhile, the effect of social relations such as place of origin, place of birth, place of first work, and other social capital factors of officials in local government behavior have also received increasing attention from scholars. For example, Jia et al. (2015) find that current top officials in China tend to have some social ties to the previous generation of top leaders and a prior history of low-level officials. Fisman et al. (2020) investigate the role of social capital such as origin and alumni in the promotion of officials and find that the promotion probability of officials with the exact origin and alumni ties as top officials is 5–9 percent. It is an unspoken rule of the bureaucracy that local government officials with social capital to government officials at a higher level are more likely to be promoted (Chu et al., 2021; Jia et al., 2021). And social capital helps develop local government officials’ loyalty to higher-ranking officials and assists higher-ranking authorities in assessing the competency of promoted officials and learning more about the local economy.

Previous studies have not paid sufficient attention to the long-term impact of policy uncertainty and the social capital of local government officials on the ETR and the sustainability of enterprises. What impact does uncertainty caused by official changes and officials’ social capital have on the ETR of enterprises, and what is the impact of enterprises’ ETR on their long-term sustainability? Our manuscript aims to provide answers to these questions.

The rest of this manuscript is organized as follows: section “Literature Review and Research Hypothesis” provides a literature review; section “Research Design” presents the data and identification strategy; section “Empirical Results and Analysis” reports the main empirical results, the robustness analysis, the mechanism analysis, and the heterogeneity analysis; section “Conclusion” concludes.

## Literature Review and Research Hypothesis

Local officials in China have played a crucial role in promoting economic system reform, attracting investment, developing the private economy, promoting exports, and improving infrastructure, among other things. Current research has not thoroughly summarized how the changes in government officials and officials’ social capital can affect the ETR of enterprises, which are the focuses of our study. Here we first investigate the previous research on the effect of policy uncertainty and officials’ social capital on the ETR of enterprises and then give some hypotheses to test.

### Change of Officers and Effective Tax Rate of Enterprises

The effective tax rate (ETR) of enterprises reflects the fairness and efficiency of the tax law in a macroscopic meaning. It is also one of the main tools for the government to formulate and implement macroeconomic policies, which helps the government achieve its macroeconomic goals. In a microscopic meaning, tax incentives are essential tools for local governments to attract foreign investment and the ETR of an enterprise also profoundly affects the corporate performance. This also makes the change of ETR become one of the focuses of corporate tax research (Spooner, 1986; Porcano, 1986; Gupta and Newberry, 1997; Dyreng et al., 2017; Drake et al., 2020). Except for corporate investment and economic performance (Ohrn, 2018; Lin and Jia, 2019), changes in the effective corporate tax rate also influence the internal factor demand of firms (Auerbach, 2018; Kim and Park, 2021), corporate growth (Ftouhi et al., 2015), corporate innovation (Cai et al., 2018; Li et al., 2021), and even entrepreneurs’ charitable donations (Baker and Dawson, 2020). At the same time, as an essential tool, the ETR is closely related to the taxing behavior of the government. Many scholars have begun to explore its influence on the ETR regarding government size, regional competition, and corporate ownership (Zhang, 2008; Wu et al., 2012; Sineviciene and Railiene, 2015; Dang et al., 2019). In reality, the lack of legislative regulation of local governments in China makes local governments use lower tax collection to attract foreign investment when facing fierce regional competition (Tang, 2020). In this manuscript, we aim to investigate the effect of policy uncertainty on enterprises’ ETR due to the change of officials, contributing to the literature on policy uncertainty and fiscal policy choices of local officers.

### Social Capital of Officials

Social capital refers to the connections between individuals or groups and social networks. It is the interpersonal connections that exist within the fabric of human relationships. Like physical and human capital, social capital connects an individual and others in an organization that can bring them future benefits (Putnam, 2001). The amount of social capital depends mainly on an individual’s social network (Borgatti et al., 1998; Burt, 2000). Putnam (1993) first introduced the concept of social capital into political science from a macro perspective. Thus, as an investment in social relations that is expected to be rewarded in the market, social capital has three main aspects: First, social capital is a resource that reflects interpersonal connections with others in the organization. In the long run, it reflects the size of the benefits. Second, social capital does not exist independently but is embedded in social relations. Individuals can interact homogeneously and heterogeneously through their social networks to access these structurally different embedded resources. Third, interaction can enhance the effect of purposeful action. Currently, academic research on social capital has shifted from theoretical analysis to empirical research by quantifying social capital and using econometric methods to examine the role of social capital on economic growth (Thompson, 2018), technological innovation (Landry et al., 2002), human capital (Mishra, 2020), and enterprise development (Habersetzer et al., 2019). However, the existing literature about the impact of officials’ social capital on corporate ETR and the sustainable development of the enterprises is inadequate. It will be one of the issues investigated in our manuscript.

### Theoretical Analysis and Research Hypothesis

The change of local officials has become a regular and institutionalized political activity. It implies the policy uncertainty of the local government due to the political instability that can have a direct impact on the sustainable development of the enterprises.

As a Chinese proverb says, “A new broom sweeps clean.” When local government officials take office, they are motivated to prove their competence by starting aggressive fiscal expansion activities and generating rapid economic growth (Chen et al., 2005; Akhtari et al., 2022). From the perspective of local officials, the competition for promotion among officials generates short-term behavior. It takes at least 2–3 years for local governments to implement their development strategy (Lv and Bai, 2019; Chen, 2021). And local officials are usually transferred to another place within 2–3 years, leading to a tendency for officials to mobilize all the resources at their disposal for a short period at the beginning of their term of office (Luo and Qin, 2021). To meet their own fiscal needs, local governments will increase their tax collection from enterprises and raise the ETR of enterprises to raise funds for the fiscal expansion they will undertake. Based on the above analysis, this manuscript puts forward the following hypothesis 1:

*Hypothesis 1: Policy uncertainty caused by changes in officials will raise the ETR of enterprises.*

Meanwhile, as another Chinese proverb says, “It is easy to be an official if you have friends at court,” and it is an unspoken rule of the bureaucracy that local government officials with social capital to government officials at a higher level are more likely to be promoted (Chu et al., 2020; Fisman et al., 2020). In this process, the social capital of local officials plays three roles: firstly, it ensures the loyalty of the promoted officers to the higher-level government officers; secondly, it helps the higher-level government official to know more about the competence of the promoted official; thirdly, through the social capital with local officials, leaders at higher levels can get more information about the local economy. Therefore, the higher level of government tends to choose local government officials who are competent and have social capital. And local officials with social capital to higher-level leaders have a greater incentive to expand fiscally to achieve their own promotion goals, which also results in an expansion of local government fiscal needs that results in a higher level of ETR of enterprises. Based on the above analysis, the following hypothesis 2 is formulated:

*Hypothesis 2: Local government officials with social capital have a greater incentive to raise the ETR of enterprises.*

And we can expect that local government officials with social capital have a greater incentive to raise the ETR of enterprises when they take office. They are more likely to be promoted and are much urgent to stimulate the economy to achieve their promotion goals. Based on the above analysis, the following hypothesis 3 is formulated:

*Hypothesis 3: Local government officials with social capital have a greater incentive to raise the ETR of enterprises when they take office.*

The data selected in this manuscript is from the Chinese Industrial Enterprise Database. Compared with the data from listed companies, the database of Chinese Industrial Enterprises contains more samples and richer types of enterprises, and these non-listed companies have a higher probability which is influenced by local government officials, which is more representative of measuring the impact of policy uncertainty caused by changes in government officials on the ETR of enterprises.

Following the research on policy uncertainty and social capital, this manuscript aims to the effect of policy uncertainty and official social capital on enterprises’ ETR due to the change of officials. Compared to previous studies, this manuscript contributes to the literature as follows. First, this manuscript investigates the impact of policy uncertainty caused by the change of local officials on enterprises at the prefectural level. This enriches the study of policy uncertainty. Second, the effect of policy uncertainty due to official change on the ETR of firms is investigated; this extends the existing studies on the evolution of tax policies of local governments (Mendoza and Oviedo, 2006; Cai and Liu, 2009; Caldara et al., 2020). Third, we expand the research on the social capital of local officers. When local government officers change tax policies after taking office, it is also of interest to examine the role of social capital in the process of policy change at this time. Above all, the research in this manuscript contributes to a better understanding of the role of officials’ social capital in local government fiscal policy decisions.

## Research Design

### Sample Selection and Data Sources

The corporate data in this manuscript comes from the Chinese Industrial Enterprise Database (1998–2009), which includes detailed information on corporate financing. We excluded enterprises with total assets less than fixed assets, fixed assets less than 1 million yuan, less than 30 workers, enterprises founded before 1949, and with an effective corporate tax rate below 0 or more than 1. The data of officials are mainly sourced from the China Research Data Service Platform (CNRDS), which contains rich data about the personal characteristics of municipal party secretaries, such as age, education, and tenure information. The socio-economic data is mainly sourced from the Wind database, which contains rich data on urban economic development, including GDP, population size, government financial status, etc. The final panel data that meets the needs of the empirical study of this manuscript is formed. We finally obtained panel data with information on government officials, firms, and local economies from 1998 to 2009.

### Model Setting and Variable Description

According to the theoretical hypotheses stated in the preceding section, the specific models of this manuscript are designed as follows:


(1) $$\begin{array}{c} \text{ln}et1_{it}=\alpha +\alpha_{1}PU_{it}+\beta X_{it}+\sum year+\sum ind+\\ \;\sum city+\varepsilon_{it} \end{array}$$

(2) $$\begin{array}{c} \text{ln}etr1_{it}=\alpha +\alpha_{1}SC_{it}+\beta X_{it}+\sum year+\sum ind+\\ \;\sum city+\varepsilon_{it} \end{array}$$

(3) $$\begin{array}{c} \text{ln}etr1_{it}=\alpha +\alpha_{1}PU_{it}+\alpha_{2}SC_{it}+\alpha_{3}PU_{it}*SC_{it}+\beta X_{it}+\\ \;\sum year+\sum ind+\sum city+\varepsilon_{it} \end{array}$$

where ln*etr*1_*it*_ is the logarithm of the ETR level of enterprise *i* in year *t*. Here, we use the value of corporate income tax/total corporate profit to represent the ETR of an enterprise. *PU*_*it*_ denotes the dummy variable for the time when the local official (municipal secretary) took office; we take the value of the time when the local official (municipal secretary) took office to be 1, otherwise it is 0. *SC*_*it*_ represents the social capital of officials, and it is a set of dummy variables; The social capital of officials includes three types: common place of origin, common first place of work, and common first school of education. We take the value of 1 if a local official and the provincial official in the province where he or she is in office have common place of origin, common first place of work, or common first school of education, and 0 otherwise. *X*_*it*_ is a group of control variables, including corporate characteristics variables: roa of the corporate (roa), the scale of fixed asset of the corporate (asset), the debt ratio of the corporate (debtratio), and the loan level of the corporate (loan); regional socioeconomic variables: the GDP of the sample city (gdp), the average wage of employed persons (income), the deficit of the sample city (deficit), the administrative area of the sample city (area), the FDI of the sample city (fdi_actual), the area of the road in the sample city (road_area), and the fixed asset investment of the city (fix_invest); and personal characteristics variables: the gender of the municipal party secretary (gender) and the level of education of the municipal party secretary (education), ∑*year*, ∑*ind*, and ∑*city* represent the year fixed effect, the industry fixed effect and region fixed effect, respectively. The variable definitions are given in Table 1.

**TABLE 1:** Variable definitions.

	Variable	Definition
Dependent variable	etr1	Corporate income tax/total profit of corporate
Independent variables	PU	Change of local officers
	SC	Social capital of local officers
Control variables	roa	Total profit of corporate/total assets of corporate
	asset	The logarithm of the scale of fixed assets of the corporate
	debt	The logarithm of Corporate interest expenditure/total asset
	loan	The logarithm of Corporate liability/total asset of corporate
	gdp	The logarithm of GDP of the sample city
	income	The logarithm of the average wage of employed persons
	deficit	The logarithm of the deficit of the sample city
	area	The logarithm of the administrative area of the sample city
	fdi	The logarithm of the FDI of the sample city
	road	The logarithm of the area of the road in the sample city
	invest	The logarithm of the fixed asset investment of the sample city
	gender	The gender of the municipal party secretary
	education	The level of education of the municipal party secretary

### Descriptive Statistical Analysis

The descriptive statistics of the variables are shown in Table 2. As can be seen, the mean of enterprises’ effective income tax rate during the sample period is 13.6%, which is much lower than the statutory rate. The lower tax rate level gives local government officials more room for discretionary fiscal policy decisions. PU is the time variable of the municipal party secretary taking office; for example, if a municipal party secretary took office in 2000, the value is 1 in 2000 and 0 otherwise.

**TABLE 2:** Descriptive statistics of the main variables.

Variable	Mean	S.D.	*Min*.	Max.	*N*
etr1	0.136	0.165	0.000	0.756	526,722
PU	0.266	0.442	0.000	1.000	526,722
SC	0.231	0.180	0.000	1.000	526,722
roa	0.047	0.186	–0.762	0.208	526,722
asset	8.506	1.592	5.826	12.998	526,722
debt	–0.730	0.666	–2.426	0.368	526,722
loan	–4.492	1.234	–7.233	–1.916	526,722
gdp	4.664	0.943	2.995	6.677	526,722
income	9.539	0.380	8.848	10.247	526,722
deficit	7.886	0.866	5.997	9.563	526,722
area	9.095	0.673	7.597	10.682	526,722
fdi	5.887	1.673	2.613	8.825	526,722
road	2.599	0.928	0.948	4.495	526,722
invest	10.604	1.148	8.428	12.609	526,722
gender	0.018	0.134	0.000	1.000	526,722
education	0.230	0.421	0.000	1.000	526,722

## Empirical Results and Analysis

### Basic Regression Results

#### The Impact of Policy Uncertainty on the Effective Tax Rate of Enterprises

First, we discuss the impact of policy uncertainty on the ETR. The main results of equation (1) are presented in Table 3. We introduce control variables and control for different fixed effects levels.

**TABLE 3:** Impact of official assignment on the effective corporate tax rate.

	(1)	(2)	(3)	(4)

	ETR	ETR	ETR	ETR
Variables	lnetr1	lnetr1	lnetr1	lnetr1
PU	−0.004**	0.003	0.003	0.020***
	(0.002)	(0.003)	(0.003)	(0.003)
roa		−0.141***	−0.141***	−0.151***
		(0.001)	(0.001)	(0.001)
asset		−0.056***	−0.056***	−0.053***
		(0.001)	(0.001)	(0.001)
debt		−0.010***	−0.010***	−0.012***
		(0.003)	(0.003)	(0.003)
loan		0.041***	0.041***	0.031***
		(0.001)	(0.001)	(0.001)
gdp		0.003	0.003	0.070***
		(0.007)	(0.007)	(0.024)
income		0.183***	0.183***	0.493***
		(0.010)	(0.010)	(0.028)
deficit		−0.027***	−0.027***	0.015***
		(0.002)	(0.002)	(0.003)
area		−0.012***	−0.012***	−0.086*
		(0.004)	(0.004)	(0.048)
fdi		−0.066***	−0.066***	−0.017***
		(0.002)	(0.002)	(0.004)
road		−0.037***	−0.037***	0.004
		(0.004)	(0.004)	(0.009)
invest		0.150***	0.150***	−0.053***
		(0.006)	(0.006)	(0.012)
gender		−0.144***	−0.144***	−0.084***
		(0.011)	(0.011)	(0.014)
education		−0.007*	−0.007*	−0.030***
		(0.004)	(0.004)	(0.006)
Constant	−1.666***	−4.052***	−4.052***	−5.263***
	(0.001)	(0.112)	(0.112)	(0.511)
Observations	526,722	526,722	526,722	526,722
*R*-squared	0.021	0.075	0.075	0.110
Year effect	Yes	Yes	Yes	Yes
Ind effect	Yes	Yes	Yes	Yes
City effect	No	No	No	Yes

***,**, and * indicate significance at the 1, 5, and 10% levels, respectively.

Specifically, we find a sound and significant positive effect of local officials’ promotion incentives on the ETR of industrial enterprises in their jurisdictions, as shown in column (4) of Table 3, which shows that the change of officials increases the ETR of industrial enterprises in their jurisdictions by 2.0% on average. The econometric regression results remain robust after controlling for official variables such as gender and education. The results of the empirical analysis show that: under the promotion system, local government officials have a strong incentive to develop the economy to gain political advancement. As a result, local officials are encouraged to take every measure to achieve better economic performance, including increased tax collection from enterprises for government investment, to improve the economic performance during their term of office, resulting in a higher level of ETR of enterprises. This verifies Hypothesis 1.

#### Impact of Social Capital on the Effective Tax Rate of Enterprises

This part discusses the impact of social capital on the ETR. The main results of equation (2) are presented in Table 4. Further, we introduce some control variables and thus control for different fixed effects levels.

**TABLE 4:** Impact of social capital of officials on the ETR of enterprises.

	(1)	(2)	(3)	(4)

	ETR	ETR	ETR	ETR
Variables	lnetr1	lnetr1	lnetr1	lnetr1
SC	0.060***	0.049***	0.049***	0.023***
	(0.003)	(0.005)	(0.005)	(0.005)
Roa		−0.142***	−0.142***	−0.151***
		(0.001)	(0.001)	(0.001)
Asset		−0.055***	−0.055***	−0.053***
		(0.001)	(0.001)	(0.001)
Debt		−0.010***	−0.010***	−0.012***
		(0.003)	(0.003)	(0.003)
Loan		0.041***	0.041***	0.031***
		(0.001)	(0.001)	(0.001)
Gdp		0.005	0.005	0.064***
		(0.007)	(0.007)	(0.024)
income		0.166***	0.166***	0.476***
		(0.010)	(0.010)	(0.029)
deficit		−0.028***	−0.028***	0.014***
		(0.002)	(0.002)	(0.003)
Area		−0.017***	−0.017***	−0.075
		(0.004)	(0.004)	(0.048)
Fdi		−0.064***	−0.064***	−0.018***
		(0.002)	(0.002)	(0.004)
Road		−0.041***	−0.041***	0.005
		(0.004)	(0.004)	(0.009)
invest		0.152***	0.152***	−0.049***
		(0.006)	(0.006)	(0.012)
gender		−0.138***	−0.138***	−0.081***
		(0.011)	(0.011)	(0.014)
education		−0.009**	−0.009**	−0.032***
		(0.004)	(0.004)	(0.006)
Constant	−1.674***	−3.878***	−3.878***	−5.188***
	(0.001)	(0.113)	(0.113)	(0.512)
Observations	526,722	526,722	526,722	526,722
*R*-squared	0.021	0.075	0.075	0.110
Year effect	Yes	Yes	Yes	Yes
Ind effect	Yes	Yes	Yes	Yes
City effect	No	No	No	Yes

***, **, and * indicate significance at the 1, 5, and 10% levels, respectively.

The results in Table 4 show that the social capital of officials has a stable and significant positive effect on the productivity of industrial enterprises. The column (4) shows that the social capital of officials increases the ETR of industrial enterprises in the jurisdiction by 2.3% on average. The econometric regression results remain robust after controlling for official variables such as gender and education. The results of the empirical analysis show that: local officials with social capital to government officials at a higher level are more likely to be promoted. They, therefore, have an incentive to demonstrate their competence by promoting rapid local economic growth through fiscal expansion, which exposes local governments to huge financing needs and leads to higher ETR of enterprises. This verifies Hypothesis 2.

#### The Boosting Role of Social Capital

In this part, we discuss the interaction effect of policy uncertainty and social capital on the ETR. The main results of equation (3) are presented in Table 5. Similarly, we introduce some control variables to control different levels of fixed effects on the findings.

**TABLE 5:** Interaction of official change and official social capital on effective corporate tax rate.

	(1)	(2)	(3)	(4)

	ETR	ETR	ETR	ETR
Variables	lnetr1	lnetr1	lnetr1	lnetr1
PU	0.016***	0.017***	0.020***	−0.015
	(0.002)	(0.003)	(0.004)	(0.017)
SC	0.038***	0.016***		
	(0.004)	(0.006)		
PU*SC	0.001	0.024***		
	(0.005)	(0.009)		
roa		−0.151***	−0.217***	−0.233***
		(0.001)	(0.002)	(0.006)
asset		−0.053***	−0.032***	−0.063***
		(0.001)	(0.003)	(0.009)
debtratio		−0.012***	0.000	0.058***
		(0.003)	(0.004)	(0.012)
loan		0.031***	0.029***	0.035***
		(0.001)	(0.002)	(0.006)
gdp		0.068***	0.282***	−0.471***
		(0.024)	(0.031)	(0.117)
income		0.473***	0.224***	1.519***
		(0.029)	(0.036)	(0.180)
deficit		0.014***	0.001	−0.001
		(0.003)	(0.004)	(0.043)
area		−0.076	−0.059	9.279***
		(0.048)	(0.115)	(1.608)
fdi		−0.017***	−0.022***	−0.087***
		(0.004)	(0.004)	(0.021)
road		0.003	−0.008	−0.407***
		(0.009)	(0.011)	(0.077)
invest		−0.047***	0.011	0.161
		(0.012)	(0.013)	(0.103)
gender		−0.082***	−0.081***	−0.016
		(0.014)	(0.016)	(0.020)
education		−0.030***	−0.035***	−0.051
		(0.006)	(0.007)	(0.053)
Constant	−1.675***	−5.196***	−5.120***	−25.075
	(0.001)	(0.512)	(1.124)	(0.000)
Observations	526,722	526,722	65,407	461,315
*R*-squared	0.057	0.110	0.082	0.073
Year effect	Yes	Yes	Yes	Yes
Ind effect	Yes	Yes	Yes	Yes
City effect	Yes	Yes	Yes	Yes

***, **, and * indicate significance at the 1, 5, and 10% levels, respectively.

In particular, the column (2) of Table 5 shows that the interaction effect of policy uncertainty caused by official changes and social capital of officials on the ETR of enterprises is significantly positive (2.4%), and the econometric regression results remain robust after controlling for official variables such as gender and education. Meanwhile, columns (3) and (4) are based on the results of group regressions with and without social capital, respectively. The results obtained from the grouped regressions show that local government officials with social capital tend to improve the ETR of enterprises. The results of the empirical analysis show that: local officials with social capital to government officials at a higher level are more likely to raise the ETR of enterprises when they take office, for they are more likely to be promoted and are much urgent to stimulate the economy to achieve their promotion goals. This verifies Hypothesis 3.

### Robustness Test

#### Explained Variables

First, we refer to Johnson (1949) and Burbidge et al. (1988) for the IHS transformation of the dependent variable (etr2), the results are shown in Table 6 below. Meanwhile, columns (4) and (5) of Table 6 are the results of group regressions with and without social capital, respectively.

**TABLE 6:** Results of regression analysis after IHS transformation for the ETR.

	(1)	(2)	(3)	(4)	(5)

	ETR	ETR	ETR	ETR	ETR
Variables	lnetr2	lnetr2	lnetr2	lnetr2	lnetr2
PU	0.020***		0.016***	0.020***	−0.017
	(0.003)		(0.003)	(0.004)	(0.016)
SC		0.022***	0.016***		
		(0.005)	(0.006)		
PU*SC			0.023**		
			(0.009)		
Constant	−5.211***	−5.138***	−5.146***	−5.091***	−99.259***
	(0.505)	(0.506)	(0.506)	(1.115)	(14.968)
Observations	526,722	526,722	526,722	65,407	461,315
*R*-squared	0.109	0.109	0.109	0.081	0.072
Controls	Yes	Yes	Yes	Yes	Yes
Year effect	Yes	Yes	Yes	Yes	Yes
Ind effect	Yes	Yes	Yes	Yes	Yes
City effect	Yes	Yes	Yes	Yes	Yes

***, **, and * indicate significance at the 1, 5, and 10% levels, respectively.

As can be seen from Table 6 above, after the replacement of the measure of the dependent variable, the results of the empirical analysis are consistent with the empirical findings of our original model.

#### Regression Analysis After Excluding Thirty-Five Large and Medium-Sized Cities

Considering larger cities tend to have higher political and economic status, which affects the likelihood of promotion of local government officials. We conduct regression analysis after excluding 35 medium and large cities from the original data sample.^1^ The results are shown in Table 7. Meanwhile, columns (4) and (5) of Table 7 are based on the results of group regressions with and without social capital, respectively.

**TABLE 7:** Results of regression analysis after excluding 35 large and medium-sized cities.

	(1)	(2)	(3)	(4)	(5)

	ETR	ETR	ETR	ETR	ETR
Variables	lnetr1	lnetr1	lnetr1	lnetr1	lnetr1
PU	0.011***		0.007*	0.008**	−0.011
	(0.004)		(0.004)	(0.004)	(0.019)
SC		0.034***	0.027***		
		(0.006)	(0.006)		
PU*SC			0.021**		
			(0.009)		
Constant	−6.787***	−6.560***	−6.572***	−5.735***	−16.868
	(0.578)	(0.581)	(0.581)	(1.022)	(14.196)
Observations	415,051	415,051	415,051	356,921	58,130
*R*-squared	0.108	0.108	0.108	0.107	0.126
Controls	Yes	Yes	Yes	Yes	Yes
Year effect	Yes	Yes	Yes	Yes	Yes
Ind effect	Yes	Yes	Yes	Yes	Yes
City effect	Yes	Yes	Yes	Yes	Yes

***, **, and * indicate significance at the 1, 5, and 10% levels, respectively.

As can be seen, the results of the empirical analysis are consistent with the empirical findings of our original model after excluding 35 medium and large cities from the original data sample.

#### Regression Analysis Excluding the Effects of the 2008 Tax Reform and the Financial Crisis

To avoid the influence of other reforms in the same period that would invalidate the results explored in this manuscript, we further sort out the different policies during the sample period.

1. Enterprise income tax reform in 2008: In 2008, China implemented the tax reform that reduced enterprises’ statutory income tax rate from 33 to 25%. The tax policy reform may affect the robustness of our regression analysis results.
2. The global economic crisis in 2008: After the global financial crisis broke out in 2008, the central government promoted a huge economic plan to stimulate economic development, which greatly impacted enterprises and local governments.

We use the subsample ranging from 1998 to 2007 to exclude the effects of the 2008 tax reform and the financial crisis. The results are shown in Table 8. Meanwhile, columns (4) and (5) of Table 8 are based on the results of group regressions with and without social capital, respectively.

**TABLE 8:** Results of regression analysis for the restricted sample interval from 1998 to 2007.

	(1)	(2)	(3)	(4)	(5)

	ETR	ETR	ETR	ETR	ETR
Variables	lnetr1	lnetr1	lnetr1	lnetr1	lnetr1
PU	0.039***		0.034***	0.037***	−0.067***
	(0.004)		(0.004)	(0.004)	(0.025)
SC		0.047***	0.032***		
		(0.007)	(0.009)		
PU*SC			0.011**		
			(0.004)		
Constant	−2.820***	−3.416***	−3.115***	−7.428***	41.531**
	(0.606)	(0.608)	(0.610)	(1.006)	(19.730)
Observations	327,807	327,807	327,807	284,215	43,592
*R*-squared	0.101	0.101	0.101	0.100	0.117
Controls	Yes	Yes	Yes	Yes	Yes
Year effect	Yes	Yes	Yes	Yes	Yes
Ind effect	Yes	Yes	Yes	Yes	Yes
City effect	Yes	Yes	Yes	Yes	Yes

***, **, and * indicate significance at the 1, 5, and 10% levels, respectively.

As shown in Table 8 above, the results of the empirical analysis are consistent with the empirical findings of our original model. This ensures the robustness of the results of our model.

### Mechanisms Analysis

#### The Role of Promotion Incentives for Officials

When new officials take office, to show their talent, they often implement large-scale economic stimulus measures to boost the economy and improve people’s living standards, which is reflected in the increase of local governments’ fiscal deficit (lndeficit) at the beginning of the officials’ tenure (Zhang and Qian, 2021). As shown in Table 9. The result in column (1) of Table 9 indicates that the new officials’ taking office (PU) leads to a 4.4% increase in the deficit of the local government.

**TABLE 9:** Impact of official assignment on local fiscal deficits.

	(1)	(2)	(3)	(4)	(5)

	Fiscal deficit	Land premium	Asset profitability	Long-term investment	Export
Variables	lndeficit	lnbargin_money	lnroa	lninvest	lnexport
PU	0.044***	0.075***			
	(0.001)	(0.007)			
lnetr1			−0.260***	−0.018***	−0.018***
			(0.002)	(0.006)	(0.003)
Constant	2.665***	−9.349***	7.335***	−6.217***	−6.311***
	(0.176)	(0.128)	(0.116)	(0.148)	(0.235)
Observations	1,450,817	1,063,720	813,130	114,883	213,394
*R*-squared	0.467	0.140	0.078	0.112	0.172
Controls	Yes	Yes	Yes	Yes	Yes
Year effect	Yes	Yes	Yes	Yes	Yes
Ind effect	No	Yes	Yes	Yes	Yes
City effect	Yes	Yes	Yes	Yes	Yes

***, **, and * indicate significance at the 1, 5, and 10% levels, respectively.

This is mainly because: (1) immediately after taking office, local government officials will adopt policies such as increasing local spending to perform better to improve their chances of promotion. (2) Because of the lagging effect of investment in many projects, it is so urgent for officials to seize the time to take such radical economic measures at the beginning of the term. (3) The average term of the officials at the prefecture-level in China is less than 3 years, so they are more focused on short-term economic growth than long-term (Li, 2018; Xi et al., 2018). When facing the increase in fiscal deficit, local government officials raised the ETR of corporates to meet their own financing needs. The same goes for land transfers (lnbargin_money), shown in column (2) of Table 9. This is mainly because (1) immediately after taking office, considering the impact of promotion incentives, local government officials will take actions such as increasing investment and fiscal expenditures to improve their promotion probability; (2) as the investment and economic effects of many projects have a lagging nature, it is so urgent to seize the opportunity to take such radical economic measures at the beginning of the tenure of local officers; (3) the rapidly expanding fiscal deficit shows a drastic need for financing, local governments then have an incentive to take measures such as increasing tax collection, raising the ETR of enterprises and land concessions to meet their own financial needs.

#### Long-Term Effects of CorpoRate Tax Rate Fluctuations

In addition to the role of promotion incentives for officials, the negative impact of the higher tax burden on the performance of the enterprises is also a consideration for local government officials to reduce corporate tax collection in the second half of their terms. However, tightened tax enforcement is a double-edged sword and can hardly last long.

Unfavorable changes in tax enforcement may drive out business activities, as shown in columns (3–5) of Table 9 above.

The results of the empirical analysis show that an increase in the effective corporate tax rate will reduce corporate profitability, depress the amount of corporate investment, and adversely affect the rise in corporate exports. The improvement of the overall economic indicators of enterprises will have a positive impact on the overall economy. Therefore, the tax increase by officials after taking office will hurt enterprises and socio-economic development. Then why do officials still invest in enterprises by increasing tax levies? We believe it is mainly because of the increase in fixed-asset investment during the officials’ new term of office and the pressure from the fiscal gap. In terms of the economic and social benefits of long-term enterprise development and the importance of increased investment in regional development, local government officials, after satisfying the short-term financing needs at the beginning of their tenure, will also consider reducing the tax levies on enterprises, fostering a favorable environment for the sustainable development of local enterprises, and promoting the regional economy.

### Heterogeneity Analysis

#### Heterogeneity Analysis According to Different Types of Social Capital

Table A1 in Appendix shows the regression analysis of different types of social capital: a common place of origin (same_nativep), the common first place of work (same_workp), and common first-degree school (same_edu_f). Government officials who have a common place of origin with provincial officials are more motivated to raise the ETR of industrial enterprises. In contrast, the effect of the common first place of work factor is insignificant, and the common first educational school factor mainly plays a negative role.

From the results of Table A1, it can be seen that hometown relations (common place of origin) have a much higher priority in the bureaucratic ranking than colleague relations (common first place of work) and classmate relations (common first school of education).

#### Heterogeneity Analysis According to Enterprise Factor Intensity

Factor intensity refers to the ratio between the factors of production required to produce different goods. Factor intensity of enterprises is also an important aspect of enterprise heterogeneity. We classify industrial enterprises into labor-intensive, capital-intensive, and technology-intensive industries according to their industries. The regression results are shown in Table A2 in Appendix.

The regression results in Table A2 show that the ETR level of enterprises in labor-intensive industries, capital-intensive industries, and technology-intensive industries is subject to a more significant factor of change by local government officials compared to that of labor-intensive industries. This is because: (1) compared to local labor employment, the improvement of the ruling performance represented by the output value of local enterprises and even regional GDP is an important issue of urgent concern for local government officials and has a higher priority in the sequence of concerns of local officials, which is also an objective requirement for the promotion tournament of officials; (2) compared to labor-intensive industries, capital-intensive industries, and technology-intensive industries, enterprises tend to have a larger enterprise scale, higher initial investment, and higher tax contribution to are one of the primary sources of tax revenue for local governments, so their ETR levels are also more likely to be affected by the change of officials factor.

## Conclusion

The political environment has a significant impact on the sustainable development of enterprises. As the legal representatives of government power, local officials can influence the economic policies, thus playing a crucial role in the development of the enterprises. This manuscript empirically examines the effect of policy uncertainty and social capital of officers on the ETR of enterprises. The main conclusions are:

First, the policy uncertainty caused by local government officials’ change significantly increases the enterprises’ ETR. Meanwhile, municipal officials who have social ties with provincial officials in their province also tend to raise the ETR of industrial enterprises.

Second, the interaction effect of policy uncertainty and the social capital of local officials are positive. It means the local officials who have social ties with provincial officials have more incentive to raise the ETR of industrial enterprises when taking office.

Third, further research show that the effect of political uncertainty on the ETR is heterogeneous, i.e., the effect on the ETR is more pronounced for enterprises in the capital-intensive and technology-intensive industries, and in addition, it has been proved that the different types of social capital affect officials’ fiscal decisions differently.

The findings of this manuscript are informative in understanding the impact of policy uncertainty on the ETR of firms and the role played by the social capital of officials in this process. These also provide the basis for measures to strengthen government regulations, improve incentives for officials to be promoted, and improve the policy-making and macro-control system to promote the sustainable development of enterprises.

## Data Availability Statement

The data analyzed in this study is subject to the following licenses/restrictions: The data used to support the findings of this study is available upon request. Requests to access these datasets should be directed to LW, fengzhizi@pku.edu.cn.

## Author Contributions

LW was the first person in charge of this manuscript and mainly responsible for the theoretical justification, methodological design, data collection, statistical analysis, and description of the results of this manuscript. DY was responsible for improving the whole design and the editing of the manuscript, including the presentation of conclusions, and policy recommendations. DL was mainly responsible for the refinement and improvement of the technical aspects of the manuscript. All authors contributed to the article and approved the submitted version.

## Conflict of Interest

The authors declare that the research was conducted in the absence of any commercial or financial relationships that could be construed as a potential conflict of interest.

## Publisher’s Note

All claims expressed in this article are solely those of the authors and do not necessarily represent those of their affiliated organizations, or those of the publisher, the editors and the reviewers. Any product that may be evaluated in this article, or claim that may be made by its manufacturer, is not guaranteed or endorsed by the publisher.

## Footnotes

1 The 35 medium and large cities include Beijing, Shanghai, Guangzhou, Shenzhen, Hangzhou, Nanjing, Tianjin, Chengdu, Wuhan, Qingdao, Ningbo, Xiamen, Chongqing, Dalian, Shenyang, Changsha, Xi’an, Zhengzhou, Jinan, Taiyuan, Changchun, Kunming, Hefei, Harbin, Fuzhou, Haikou, Nanchang, Shijiazhuang, Hohhot, Urumqi, Nanning, Lanzhou, Guiyang, Yinchuan, and Xining.

## Appendix

Tables A1, A2 reports the results of heterogeneity analysis according to gender and age, first-degree and first-degree major, different types of social capital, enterprise location, and enterprise factor intensity.

**TABLE A1:** Regression analysis grouped by different types of social capital.

	(1)	(2)	(3)

	ETR	ETR	ETR
Variables	lnetr1	lnetr1	lnetr1
same_nativep	0.047***		
	(0.006)		
same_workp		0.021	
		(0.040)	
same_edu_f			−0.025**
			(0.010)
Constant	−4.800***	−5.248***	−5.119***
	(0.515)	(0.511)	(0.512)
Observations	526,722	526,722	526,722
*R*-squared	0.110	0.110	0.110
Controls	Yes	Yes	Yes
Year effect	Yes	Yes	Yes
Ind effect	Yes	Yes	Yes
City effect	Yes	Yes	Yes

****, **, and * indicate significance at the 1, 5, and 10% levels, respectively.*

**TABLE A2:** Regression analysis of firms grouped by factor intensity.

	(1)	(2)	(3)

	labor	capital	technology
Variables	lnetr1	lnetr1	lnetr1
PU	−0.003	0.024***	0.023***
	(0.010)	(0.006)	(0.004)
Constant	−6.034***	−3.307***	−6.254***
	(1.466)	(0.897)	(0.713)
Observations	64,222	168,546	292,746
*R*-squared	0.109	0.107	0.118
Controls	Yes	Yes	Yes
Year effect	Yes	Yes	Yes
Ind effect	Yes	Yes	Yes
City effect	Yes	Yes	Yes

****, **, and * indicate significance at the 1, 5, and 10% levels, respectively.*
